# Diabetes and Hypertension among Patients Receiving Antiretroviral Treatment Since 1998 in Senegal: Prevalence and Associated Factors

**DOI:** 10.5402/2012/621565

**Published:** 2012-12-01

**Authors:** Assane Diouf, Amandine Cournil, Khadidiatou Ba-Fall, Ndèye Fatou Ngom-Guèye, Sabrina Eymard-Duvernay, Ibrahima Ndiaye, Gilbert Batista, Papa Mandoumbé Guèye, Pape Samba Bâ, Bernard Taverne, Eric Delaporte, Papa Salif Sow

**Affiliations:** ^1^Centre Régional de Recherche et de Formation à la Prise en charge du VIH/sida et maladies associées (CRCF), Université Cheikh Anta Diop (UCAD), BP 45690, Dakar, Senegal; ^2^Service des Maladies Infectieuses, Centre Hospitalier Universitaire (CHU) de Fann, BP 5035, Dakar, Senegal; ^3^UMI 233, Institut de Recherche pour le Développement (IRD), Université de Montpellier 1, BP 64501, 34394 Montpellier Cedex 5, Montpellier, France; ^4^Service de Médecine Boufflers, Hôpital Principal de Dakar, BP 3006, Dakar, Senegal; ^5^Centre de Traitement Ambulatoire (CTA), CHU de Fann, BP 16760, Dakar, Senegal

## Abstract

Cardiovascular risk factors in people on antiretroviral treatment (ART) are poorly documented in resource-constrained settings. A cross-sectional study was conducted in 2009 to assess prevalence of diabetes and hypertension in a sample of 242 HIV-infected patients who had initiated ART between 1998 and 2002 in Dakar, Senegal (ANRS 1215 observational cohort). World Health Organization (WHO) criteria were applied to diagnose diabetes and hypertension. Multiple logistic regressions were used to identify factors associated with diabetes and hypertension. Patients had a median age of 46 years and had received ART for a median duration of about 9 years. 14.5% had diabetes and 28.1% had hypertension. Long duration of ART (≥119 months), older age, higher body mass index (BMI), and higher levels of total cholesterol were associated with higher risks of diabetes. Older age, higher BMI at ART initiation, and higher levels of triglycerides were associated with higher risk of hypertension. This study shows that diabetes and hypertension were frequent in these Senegalese HIV patients on ART. It confirms the association between duration of ART and diabetes and highlights the need to implement programs for prevention of cardiovascular risk factors in HIV patients from resource-constrained settings.

## 1. Introduction

In December 2010, over five million people were on antiretroviral treatment (ART) in Sub-Saharan Africa [1]. In Senegal, this number reached 18,000 at the end of 2011, corresponding to a coverage rate of 78% [2].

The efficacy of ART has led to a significant reduction in mortality among people living with HIV [3–6], causing an increase in their life expectancy, which nevertheless remains below that of the general population [7–10]. This excess mortality is partially related to immunodepression but also organic and metabolic disorders that are not classified as AIDS [4, 11–16]. These disorders have multifactor causes. The underlying physiopathological mechanisms have not been clearly established. Nevertheless, it is recognized that they involve phenomena related to the virus [17–21], to antiretrovirals (ARVs) [22–26], and to the host [27–31]. These mechanisms contribute to noninfectious diseases including diabetes and hypertension, which are two major risk factors of cardiovascular disease and are associated with increased mortality and morbidity.

Available data on prevalence and the factors associated with diabetes and hypertension have dealt with populations of varying ages and whose duration of ARV exposure varied greatly. These studies have rarely addressed Sub-Saharan Africa where most countries have only recently begun ART in the 2000s. 

In 1998, Senegal was one of the first resource-constrained countries to implement a government program providing free access to ARVs. A cohort of the 403 initial patients included in this program received routine followup, thus providing an opportunity to study the long-term impact of ART. Therefore, this study was conducted with two objectives: (1) to measure the prevalence of diabetes and hypertension among patients who have been receiving ART for several years in Senegal and (2) identify the factors associated with diabetes and hypertension.

## 2. Materials and Methods

### 2.1. Study Design and Population

A cross-sectional study was conducted between December, 2009 and March 31, 2010. Patients were recruited within the ANRS 1215 observational cohort, which initially included 403 HIV-1 patients enrolled in the Senegalese Antiretroviral Drug Access Initiative between 1998 and 2002. A total of 242 patients who were still alive and receiving follow-up on December 1, 2009 at the Fann University Hospital in Dakar were enrolled in this study. All patients were receiving antiretroviral treatment. 

The study was approved by the Senegalese National Ethics Committee.

### 2.2. Definitions

Clinical and biological characteristics, including AIDS stage according to US Centers for Disease Control and Prevention (CDC) classification, hepatitis C or B coinfection, date of ART initiation, type and duration of all antiretroviral treatments received since initiation, CD4 cell count, and body mass index (BMI) at treatment initiation, were extracted from the ANRS 1215 cohort database. 

BMI, CD4 cell count, viral load, levels of total cholesterol and triglycerides at the time of the study, and diabetes and hypertension diagnostics were collected from medical and laboratory files using standardized forms.

Patients were considered diabetic if their fasting blood glucose was ≥7.0 mmol/l at two consecutive measurements or if they were known to be diabetic patients. 

Patients were considered as hypertensive if their systolic blood pressure was ≥140 mmHg and/or their diastolic blood pressure ≥90 mmHg at three consecutive measurements separated by at least two weeks and taken after a 1-hour rest in a sitting position or if they were known to be hypertensive and were treated for it.

### 2.3. Statistical Analysis

Frequencies of diabetes and of hypertension were compared according to the main characteristics of the study sample using *χ*²-tests or Fisher's exact tests when appropriate.

Logistic regression was used to identify factors associated with diabetes and with hypertension. Age, sex, current BMI, BMI at treatment initiation, CDC stage, coinfection with hepatitis C virus (HCB) or hepatitis B virus (HBV), current CD4 cell count, CD4 count at treatment initiation, viral load, total cholesterol levels, triglyceride levels, treatment duration for each type of ARV drug, and total duration of ART were included in a bivariate analysis. All variables with *P* < 0.20 in the bivariate analysis were entered in a multiple logistic regression. Treatment duration was entered as a continuous variable if the linearity of the association with the outcome variable was verified, and as a categorical variable if not. In the latter situation, the treatment duration (in months) was categorized in the following quartiles: [89–98[; [98–107[; [107–119[; [119–139[. The Hosmer-Lemeshow test was used to assess the goodness of fit of the logistic regression models. 

All statistical tests were two-sided and considered statistically significant at a 5% threshold. Statistical analyses were performed using SAS statistical software (version 9.2, Cary, NC, USA). 

## 3. Results

### 3.1. Characteristics of the Study Population

A total of 242 patients who agreed to participate were enrolled in the study. They had initiated ART between 1998 and 2002. The initial ARV regimen was a triple drug combination with two nucleoside reverse transcriptase inhibitors (NRTI) + a nonnucleoside reverse transcriptase inhibitor (NNRTI) or a protease inhibitor (PI). At treatment initiation, 39.3% received a PI-based regimen. Median treatment duration was almost 9 years. Some 75% of the patients had been receiving ART for at least 8 years. At the time of the study, half of the patients had a CD4 cell count above 500 cells/*μ*L, and viral load was undetectable for 83% (Table 1).

The most commonly prescribed NRTI combinations were lamivudine (3TC) + zidovudine (AZT) and stavudine (d4T) + didanosine (ddI). As for NNRTI and PI, the most commonly used molecules were efavirenz (EFV) and indinavir (IDV), respectively (Table 2).

### 3.2. Prevalence and Factors Associated with Diabetes

Among the 242 patients, 39 had diabetes, corresponding to a prevalence of 14.5% (95% confidence interval (CI): 10.3–19.5). The frequency of diabetes was higher in patients ≥45 years of age and in those with long duration of treatment (≥119 months) (Table 3). Those who had hypercholesterolemia or elevated triglycerides also had higher prevalence of diabetes.

Variables associated with diabetes in bivariate analyses were long duration of ART, age, BMI at treatment initiation, levels of total cholesterol and triglycerides, and duration of exposure to d4T, ddI, and IDV. In multivariate analysis, long duration to ART, older age, higher BMI at treatment initiation, and higher level of triglycerides were independently associated with higher risk of diabetes (Table 4).

### 3.3. Prevalence and Factors Associated with Hypertension

The prevalence of hypertension in the study population was 28.1% (CI: 22.5–34.2). Out of 68 patients who had hypertension (or were receiving antihypertensive treatment), 15 (22%) were aware of their condition before initiating ARV therapy. Patients over 45 years of age were more likely to have hypertension. Higher prevalence of hypertension was also observed for male patients, those who were overweight (BMI ≥ 25 kg/m²) at treatment initiation or at time of the study, those who had a CD4 count above 200 cells/*μ*L, and those presenting with hypercholesterolemia or hypertriglyceridemia, but differences were not statistically significant (Table 3). 

In multivariate analysis, older age, higher current BMI, higher cholesterolemia, and longer duration of IDV exposure were associated with higher risk of hypertension. Total duration of ART was not found to be associated, and longer exposure to lopinavir/ritonavir (LPV/r) was associated with a reduced risk of hypertension (Table 4).

## 4. Discussion

The patients in this study were black Africans who initiated ART in Senegal between 1998 and 2002 and who have received regular followup until December 1, 2009. At that date, they had been receiving ARVs for a median duration of nearly 9 years; half of the patients were over 46 years old. Prevalence of diabetes and hypertension was 14.5% and 28.1%, respectively.

After adjustment, diabetes appeared to be associated with the duration of ARV exposure while hypertension did not. Other associated factors were the same as those for the general population.

One of this study's strengths is its investigation of the long-term impact of treatments with ARV-exposure durations rarely observed in other developing countries where ART was available well after 1998. Some treatments used at inclusion, such as IDV, nelfinavir (NFV), or d4T (particularly the 40 mg dosage), are no longer in use. However, most of the patients on ARVs in developing countries were also exposed to these molecules for long durations. Our study results may be useful for resource-constrained countries that began with ARV triple therapies in the 2000s in populations that generally had the same characteristics as those in our cohort. These results provide a long-term view for these countries where ART started several years after Senegal. 

Another strength is having used definitions for diabetes and hypertension that took into account several successive measurements in accordance with WHO guidelines or those of specialized American associations. 

Interpretation of our results must take into account several limitations. This is a cross-sectional study and, as such, could only identify associated factors and not risk factors. Moreover, some potentially confounding factors were not taken into account in the analysis. Family history of diseases under study, the CD4 nadir, and adherence to ART were not collected.

After a median follow-up duration of 107 months, 14.5% of patients presented with diabetes. Data from developing countries come from three studies. A study in Benin reported a prevalence of 8% after a median exposure to ARVs of 23.2 months and an average age of 38 years [32]. In South Africa, only four cases of diabetes were identified among 304 patients exposed to ARVs for more than one year [33]. A recent study involving 4010 patients from seven countries in Latin America found an overall prevalence of 3.6% for type 2 diabetes [34]. The lower prevalence found in these studies when compared to ours can be explained in part by two factors: a younger population and, in some cases, a shorter duration of ARV exposure. 

More research in the literature has been published from developed countries, and it deals with both the prevalence and incidence of diabetes. Most of these studies report prevalence and/or incidence rates higher than observed in the general population [35–38], while others report similar [39], or even lower [34], figures. Even though conventional risk factors have been confirmed in this population, ARV exposure is not unanimously recognized as a factor associated with diabetes [36, 37, 40–46]. 

In France, data from the ANRS cohort APROCO-COPILOTE report a prevalence of 9% for diabetes after 8 years of ART [47]. In a cohort of 1278 males from the United States (Multicenter AIDS Cohort Study (MACS)), the prevalence of diabetes, adjusted for age and BMI, was 14% among seropositive patients on ARVs, corresponding to 4.6 times greater than among seronegative patients. This same study estimated a 10% risk of developing diabetes after 4 years of treatment [37]. Incidences of 2.50/100 person/years and 2.89/100 person/years, respectively, were reported in the study by Tien et al. among patients on PI-based triple therapy and those on triple therapy not containing IP [36]. In the D:A:D study, incidence was 4.42/1000 person/years [48] and 5.72/1000 for a Swiss cohort [43]. The prevalence observed in our study is higher than that reported in the French study [47] and nearly identical to the one found in the MACS. However, differences between the study populations must be taken into account, particularly in terms of age, ethnicity, and treatment duration. In France, the prevalence of impaired glucose tolerance rose significantly with age, BMI, and exposure to IDV [47]. In the MACS, the factor having a high association with the onset of diabetes was ART (HR = 4.11; CI: 1.85–9.16 after adjusting for age and BMI) [37], although it was NRTI exposure in the female population for the Tien PC study [36]. In the Swiss cohort, the factors associated with the onset of diabetes were male sex, age >60 years, African or Asian ethnicity, stage C of CDC classification, obesity, NRTI exposure, and PI exposure [43]. All of the associated factors found in our study were also found in the D:A:D study to be risk factors for diabetes [48]. A recent publication from the APROCO-COPILOTE cohort also found that advanced age, overweight, and exposure to IDV were among the risk factors for incident diabetes. This involved patients who received ARVs for a median duration of 9.6 years with an incidence of 14.1/1000 person/years [49].

Studies on hypertension among people living with HIV report a wide range of prevalence [33, 38, 50–56]. Factors associated with this prevalence are the same as those found in the general population. The role of ART remains controversial [38, 48, 52–55, 57–61].

Few studies have been conducted in the South. In Johannesburg, a prevalence of 19.1% was observed among patients infected with HIV, ages 18 to 45 years, and on ARVs for over one year [33]. In Brazil, two studies reported a prevalence of 19.9% and 25.6%, respectively [38, 50]. The study of the South American cohort reported a prevalence of 31.5% [34] but used a different definition (systolic blood pressure ≥ 130 mm Hg and/or diastolic blood pressure ≥ 85 mm Hg) than that of our study. Factors associated with hypertension were age, male sex, overweight, and hypertriglyceridemia [50].

In developed countries, prevalence ranges between 20% and 40% among populations exposed to ARVs, however, with differences in terms of age; ethnicity; duration of ARV exposure; clinical, immunological and virological status [51–56]. These prevalence figures were reported in Norway, Spain, Italy, and the United States. The role of conventional risk factors recognized in the general population was unanimously highlighted in all these studies. The role of ART was questionable. In Norway, treatment duration appeared to be a predictive factor for hypertension in one study, but it did not investigate the roles of various ARV molecules [52]. Another case-control study reported a higher prevalence of hypertension among people on ARVs compared to those who were not (21% versus 13%, resp.), but the difference was not significant [54]. There was also no difference in prevalence based on whether or not patients were exposed to NNRTIs or PIs.

In Spain, a rise in blood pressure was reported after 48 weeks of ART. The authors suggested that treatment had a partial role in raising blood pressure by improving the patient's health condition [53]. This study only involved 95 patients with a treatment duration of 48 weeks, making it difficult to draw a precise conclusion on this point. Gazzaruso et al. found no association between ART and hypertension, but their study only examined exposure to NRTIs and PIs and not the duration of treatment exposure nor each ARV molecule [55].

Thiébaut et al. investigated risk factors of the onset of hypertension in the D:A:D study and exposure to ARVs did not appear to be a risk factor for hypertension after adjustment. On the other hand, the cumulative duration of exposure to NNRTIs was associated with a slight risk of hypertension (a reduced risk between 22% and 33% was observed among those who were exposed to NNRTIs compared to those who never received any NNRTIs) [59].

In the United States, it was found that atazanavir (ATV) played a protective role in the onset of raised blood pressure compared to EFV [62]. This same study found a slight risk of a raised blood pressure among patients exposed to ATV, NFV, and IDV compared to patients exposed to LPV/r. This did not agree with our results, which found that hypertension had a positive association with IDV and a negative one with LPV/r. Nevertheless, it should be noted that this last study only took into account ART at inclusion in contrast to ours, which investigated exposure duration. On the other hand, our patients were exposed to high concentrations of IDV (2400 mg/day) without ritonavir. The association between IDV and hypertension, observed in an earlier study, is likely linked to the renal toxicity of this molecule [63]. 

Our study suggests that prevalence of diabetes and hypertension is higher among people exposed to ARVs compared to the general population in Senegal [64, 65]. A recent study reported prevalence of 10.4% and 46% for diabetes and hypertension, respectively, but the diagnosis of hypertension was based on one measure and was certainly overestimated [66]. There is a positive association between ART duration and diabetes. This observation is consistent with most of the literature that has assessed the association between ARVs and diabetes. However, the association between certain ARV molecules and systolic hypertension seems more controversial. 

Diabetes and hypertension are known risk factors for cardiovascular disease in low- and middle-income countries which represents more than 80% of the global burden of cardiovascular disease worlwide [67]. For patients on ARVs, these factors combine with other comorbidities [68, 69] and cause high mortality [70]; otherwise, they have a potentially negative impact on the quality of life and efficacy of ARVs. 

Treatment for these diseases occurs over the long term, if not for life, thus, posing problems regarding cost and observance in resource-constrained countries. This highlights the importance of implementing screening, early diagnosis, and prevention measures for these disorders.

Screening for diabetes and hypertension must be an integral part of followup for HIV-positive persons, more specifically among those who are on ARVs. The same applies for other prevention measures for cardiovascular disease (tobacco use, overweight, and hyperlipidemia). To do this, models for followup must integrate these parameters. Diet and lifestyle advice, including smoking cessation, regular physical activity, and a balanced diet, must be integrated into routine consultations for these populations. Measurements of blood pressure, waist circumference and glycemia, cholesterolemia, and triglyceridemia must also be taken regularly, at least at a certain age and/or specific duration of ARV exposure and even for a specified cardiovascular risk level. 

Our study provides the first data on diabetes and hypertension among people who are living with HIV and on ART in Senegal. This contributes to improved assessment and better analysis of this problem in the context of resource-constrained countries where literature on this topic is rare.

## Figures and Tables

**Table 1:** Demographics, HIV, and metabolic characteristics of the study population.

Characteristics	*N *	(%)/median (IQR)
Age, median years (IQR)	242	46 (40–54)
<45	98	(40.5)
≥45	144	(59.5)
Sex		
Male	102	(42.1)
Female	140	(57.9)
BMI, median kg/m² (IQR)	242	22.3 (19.8–25.6)
<25	174	(71.9)
≥25	68	(28.1)
BMI at treatment initiation, median kg/m² (IQR)	242	20.2 (18.4–22.6)
<25	217	(89.7)
≥25	25	(10.3)
CDC stage		
A	16	(6.6)
B	99	(40.9)
C	127	(52.5)
HCV or HBV infection	45	(18.6)
CD4 cell count, median cells/*μ*L (IQR)	242	501 (372–722)
≤200	13	(5.4)
>200	229	(94.6)
CD4 cell count at treatment initiation, median cells/*μ*L (IQR)	239	146 (61–229)
≤200	151	(63.2)
>200	88	(36.8)
HIV viral load < 50 copies/mL	201	(83.1)
Total cholesterol (g/L)		
<1.5	224	(92.6)
≥1.5	18	(7.4)
Triglycerides (g/L)		
<2.4	231	(95.5)
≥2.4	11	(4.5)

IQR: interquartile range; BMI: body mass index; HCV: hepatitis C virus; HBV: hepatitis B virus.

**Table 2:** Main ARV drugs used and mean duration of exposure since ART initiation.

Type of ARV drug*	Number of patients exposed	Mean duration	
		(months) (±SD)	
Any ARV	242	110	(±13)
Zidovudine (AZT)	173	52	(±45)
Stavudine (d4T)	103	23	(±33)
Didanosine (ddI)	156	50	(±48)
Lamivudine (3TC)	240	88	(±32)
Efavirenz (EFV)	182	55	(±44)
Nevirapine (NVP)	77	22	(±47)
Indinavir (IDV)	107	25	(±33)
Lopinavir/ritonavir (LPV/r)	61	7	(±15)

Abacavir, nelfinavir, and tenofovir were used by a few patients with very short durations of exposure.

**Table 3:** Prevalence of diabetes and hypertension according to demographics, HIV-related, and biological characteristics of the study population.

Characteristics	Diabetes		Hypertension	
	Frequency (%)	*P *	Frequency (%)	*P *
ART duration, months		<0.001		0.81
Short	8.3		27.4	
Long*	32.8		28.8	
Age, years		<0.001		0.002
<45	5.1		17.3	
≥45	20.8		35.4	
Sex		0.41		0.12
Male	16.7		33.3	
Female	12.9		24.3	
BMI, kg/m²		0.64		0.22
<25	13.8		25.9	
≥25	16.2		33.8	
BMI at treatment initiation, kg/m²		0.22		0.35
<25	13.4		27.2	
≥25	24.0		36.0	
CDC stage		0.63		0.53
A	12.5		18.7	
B	12.1		26.3	
C	16.5		30.7	
HCV or HBV coinfection		0.47		0.39
Yes	11.1		33.3	
No	15.2		26.9	
CD4 cell count, cells/*μ*L		0.70		0.09
≤200	7.7		7.7	
>200	14.8		29.3	
CD4 cell count at treatment initiation, cells/*μ*L		0.47		
≤200	15.9			
>200	12.5			
HIV viral load copies/mL		0.64		0.56
<50	14.9		28.9	
≥50	12.2		24.4	
Total cholesterol (g/L)		0.03		0.11
<1.5	12.9		26.8	
≥1.5	33.3		44.4	
Triglycerides (g/L)		0.01		0.53
<2.4	13.0		27.7	
≥2.4	45.4		36.4	

*Long duration of ART was defined for durations ≥119 months for comparison of diabetes prevalence and for durations ≥107 months for the comparison of hypertension.

**Table 4:** Multiple regression analyses for factors associated with diabetes and hypertension.

Characteristics	Diabetes		Hypertension	
	OR	95% CI	OR	95% CI
ART duration, months			0.99	0.96–1.01
Short < 119	1			
Long ≥ 119	3.78	1.69–8.42		
Age, years	1.06	1.01–1.11	1.06	1.02–1.10
Sex				
Male			1	
Female			0.83	0.41–1.69
BMI, kg/m²			1.10	1.03–1.18
BMI at treatment initiation, kg/m²	1.13	1.02–1.26	1.07	0.95–1.19
CD4 cell count, cells/*μ*L			1.00	0.98–1.02
Total cholesterol (g/L)	1.78	0.68–4.66	2.48	1.19–5.16
Triglycerides (g/L)	2.32	1.12–4.81	1.34	0.67–2.69
Drug exposure duration (months)				
Zidovudine (AZT)	1.05	0.97–1.13		
Stavudine (d4T)	1.06	0.99–1.15	1.01	0.95–1.08
Didanosine (ddI)	0.98	0.90–1.06		
Nevirapine (NVP)	0.99	0.92–1.06		
Indinavir (IDV)	1.03	0.95–1.13	1.10	1.03–1.16
Lopinavir/ritonavir (LPV/r)	0.84	0.67–1.05	0.84	0.71–0.99

OR: odds-ratio; CI: confidence interval; BMI: body mass index; HCV: hepatitis C virus; HBV: hepatitis B virus.

## Appendix

### The ANRS 1215 Study Group

- Ibra Ndoye (Conseil National de Lutte contre le Sida, Dakar, Sénégal),
- Eric Delaporte, Jean-François Etard, Martine Peeters, Alice Desclaux, Pierre de Beaudrap, Christian Laurent, Julie Coutherut, Tidiane Ndoye, Nicole Vidal, Claire Moquet, Sabrina Eymard-Duvernay, Cécile Cames, Kirsten Bork, Sabah Boufkhed, Mathilde Couderc, Amandine Cournil, Bernard Taverne (IRD, UMI 233; Université Montpellier 1, France).
- Assane Diouf, Mame Basty Koïta Fall, Alle Baba Dieng, Christian Eric Massidi, Adama Sarr, Khoudia Sow, Mariane Ndiaye Berthe, Saïdou Ba, Absa Ba, Catherine Lissoune Fall Sané, Sokhna Boye, Caroline Desclaux, Héléne Dior Mbodj, Coumba Gueye Cissé, Frédérique Muller, Kouro Bousso Niang, Marie-Louise Sarr, Estelle Simen, Alassane Sow, El Hadj Malick Sy Camara (Centre régional de recherche et de formation à la prise en charge, Centre Hospitalier National Universitaire de Fann, Dakar, Sénégal),
- Maryvonne Maynard, Isabelle Lanièce, Vanina Cilote (Service de Coopération et d'Action Culturelle, Ambassade de France, Dakar, Sénégal),
- Papa Salif Sow, Ibrahima Ndiaye, Cheickh Tidiane Ndour, Viviane Pierre Marie Ciss, (Centre Hospitalier National Universitaire de Fann, Service des Maladies Infectieuses et Tropicales, Dakar, Sénégal),
- Ndeye Fatou Ngom Guèye, Djibril Baal, Batista Gilbert, Andréa Robalo Diassy (Centre Hospitalier National Universitaire de Fann, Centre de Traitement Ambulatoire, Dakar, Sénégal),
- Jeanne Diaw (Hôpital Général de Grand Yoff, Dakar, Sénégal)
- René Ecochard, (Université Claude Bernard Lyon I, France)
- Mathieu Bastard (Epicentre, Paris, France)
- Kadidiatou Ba Fall, Pape Madoumbé Guèye, Pape Samba Ba, Madiouba Diawara (Hôpital Principal de Dakar, Dakar, Sénégal),
- Souleymane Mboup, Pape Alassane Diaw, Halimatou Diop Ndiaye, Ndeye Coumba Touré Kane, Moussa Thiam (Centre Hospitalier National Universitaire Le Dantec, Laboratoire de Virologie et Bactériologie, Dakar, Sénégal),
- Karim Diop (Ministère de la Santé, Division de lutte contre le sida, Dakar, Sénégal),
- Bara Ndiaye (Centre Hospitalier National Universitaire de Fann, Pharmacie centrale, Dakar, Sénégal).
